# Influence of Model Evolution and System Roles on ChatGPT’s Performance in Chinese Medical Licensing Exams: Comparative Study

**DOI:** 10.2196/52784

**Published:** 2024-08-13

**Authors:** Shuai Ming, Qingge Guo, Wenjun Cheng, Bo Lei

**Affiliations:** 1 Department of Ophthalmology, Henan Eye Hospital, Henan Provincial People’s Hospital, Zhengzhou, China; 2 Eye Institute, Henan Academy of Innovations in Medical Science, Zhengzhou, China; 3 Henan Clinical Research Center for Ocular Diseases, People’s Hospital of Zhengzhou University, Zhengzhou, China; 4 Department of Ophthalmology, People’s Hospital of Zhengzhou University, Zhengzhou, China

**Keywords:** ChatGPT, Chinese National Medical Licensing Examination, large language models, medical education, system role, LLM, LLMs, language model, language models, artificial intelligence, chatbot, chatbots, conversational agent, conversational agents, exam, exams, examination, examinations, OpenAI, answer, answers, response, responses, accuracy, performance, China, Chinese

## Abstract

**Background:**

With the increasing application of large language models like ChatGPT in various industries, its potential in the medical domain, especially in standardized examinations, has become a focal point of research.

**Objective:**

The aim of this study is to assess the clinical performance of ChatGPT, focusing on its accuracy and reliability in the Chinese National Medical Licensing Examination (CNMLE).

**Methods:**

The CNMLE 2022 question set, consisting of 500 single-answer multiple choices questions, were reclassified into 15 medical subspecialties. Each question was tested 8 to 12 times in Chinese on the OpenAI platform from April 24 to May 15, 2023. Three key factors were considered: the version of GPT-3.5 and 4.0, the prompt’s designation of system roles tailored to medical subspecialties, and repetition for coherence. A passing accuracy threshold was established as 60%. The *χ*^2^ tests and κ values were employed to evaluate the model’s accuracy and consistency.

**Results:**

GPT-4.0 achieved a passing accuracy of 72.7%, which was significantly higher than that of GPT-3.5 (54%; *P*<.001). The variability rate of repeated responses from GPT-4.0 was lower than that of GPT-3.5 (9% vs 19.5%; *P*<.001). However, both models showed relatively good response coherence, with κ values of 0.778 and 0.610, respectively. System roles numerically increased accuracy for both GPT-4.0 (0.3%‐3.7%) and GPT-3.5 (1.3%‐4.5%), and reduced variability by 1.7% and 1.8%, respectively (*P*>.05). In subgroup analysis, ChatGPT achieved comparable accuracy among different question types (*P*>.05). GPT-4.0 surpassed the accuracy threshold in 14 of 15 subspecialties, while GPT-3.5 did so in 7 of 15 on the first response.

**Conclusions:**

GPT-4.0 passed the CNMLE and outperformed GPT-3.5 in key areas such as accuracy, consistency, and medical subspecialty expertise. Adding a system role insignificantly enhanced the model’s reliability and answer coherence. GPT-4.0 showed promising potential in medical education and clinical practice, meriting further study.

## Introduction

ChatGPT, a general large language model (LLM) developed by OpenAI, has gained substantial attention since its launch on November 30, 2022. Known for its advanced natural language processing capabilities, ChatGPT has the potential to make significant impacts on many industries, including medical education. Its performance in medicine was first tested at or near the passing threshold of the United States Medical Licensing Examination (USMLE) [1,2]. While ChatGPT’s accuracy varies across languages [3], it has been tested on a series of medical exams like the Japanese National Medical Licensing Examination in languages including English [4], Chinese [5], Dutch [6], Japanese [7], and Korean [8]. The research scope related to ChatGPT has expanded to medical education in fields like nuclear medicine [9], neurosurgery [10], ophthalmology [11], general chemistry, nursing[12], life support [4], dentology [13], and radiation oncology physics [14]. Overall, while ChatGPT demonstrates heterogeneous capabilities, it shows promising potential in these medical specialties.

Several factors might influence ChatGPT’s performance. First, the updated version of ChatGPT, GPT-4, understands and generates natural language in more complex and nuanced scenarios, leading to more accurate responses [15], which is important in analyzing complex clinical case questions [16]. Thus, GPT-4 conclusively demonstrated significantly better performance than GPT-3.5, as evidenced by various official medical exams [8]. Besides the model version, ChatGPT allows users to guide its behavior by adding prompts that describe its system role. These system roles influence the direction of ChatGPT’s answers and may affect its reliability. However, the impact of these system roles on ChatGPT’s performance in medical field has not yet been investigated. As a professional chatbot tool, ChatGPT uses sampling to predict the next token with varying distribution probabilities, ensuring responses are varied and natural in real-world applications. Zhu et al [17] have found that composite answers derived from repeated questioning can enhance the accuracy of ChatGPT. Typically, 2 or 3 repeated responses are necessary to ensure response stability [18-20].

Currently, the peer-reviewed research still lacks highlights on the strength of ChatGPT when it comes to the Chinese National Medical Licensing Examination (CNMLE). This study aimed to evaluate the performance of ChatGPT in answering CNMLE questions in the clinical setting of China, with consideration of the version of ChatGPT and system role.

## Methods

### The CNMLE 2022 Question Data

As an industry admission examination, passing the CNMLE means that a medical practitioner meets the minimum medical competencies. The written part of the examination, which emphasizes medical knowledge and clinical decision-making skills, is created and supervised by the Chinese National Medical Examination Center (NMEC). In 2021, the CNMLE transitioned from the traditional paper-based format to a computer-based examination. Each candidate is presented with 600 questions, arranged in a slightly varied order, from the exam year’s question data set. According to OpenAI’s introduction, ChatGPT’s responses are based on information available up to September 2021. Thus, we selected the CNMLE 2022 questions, which were purchased from a web-based bookstore [21], for our evaluation. This choice ensured that the questions had not been previously encountered and trained by the model. The publisher has confirmed that these released questions are the original ones from the examination.

The CNMLE 2022 covered 600 single-answer multiple-choice questions, which were evenly divided into 4 units [22]. Each unit had 4 specific question types: A1, the single-sentence optimal choice questions; A2, case summary optimal choice questions; A3/A4, case group optimal choice questions; and B1, standard combination questions. Detailed explanations of each question type was conveyed to ChatGPT via a structured prompt prior to inquiry (see in Multimedia Appendix 1). The CNMLE 2022 questions did not involve table or image-based questions. Therefore, ChatGPT, despite lacking multimodal capabilities, was still suited to effectively complete the test.

**Figure 1.**
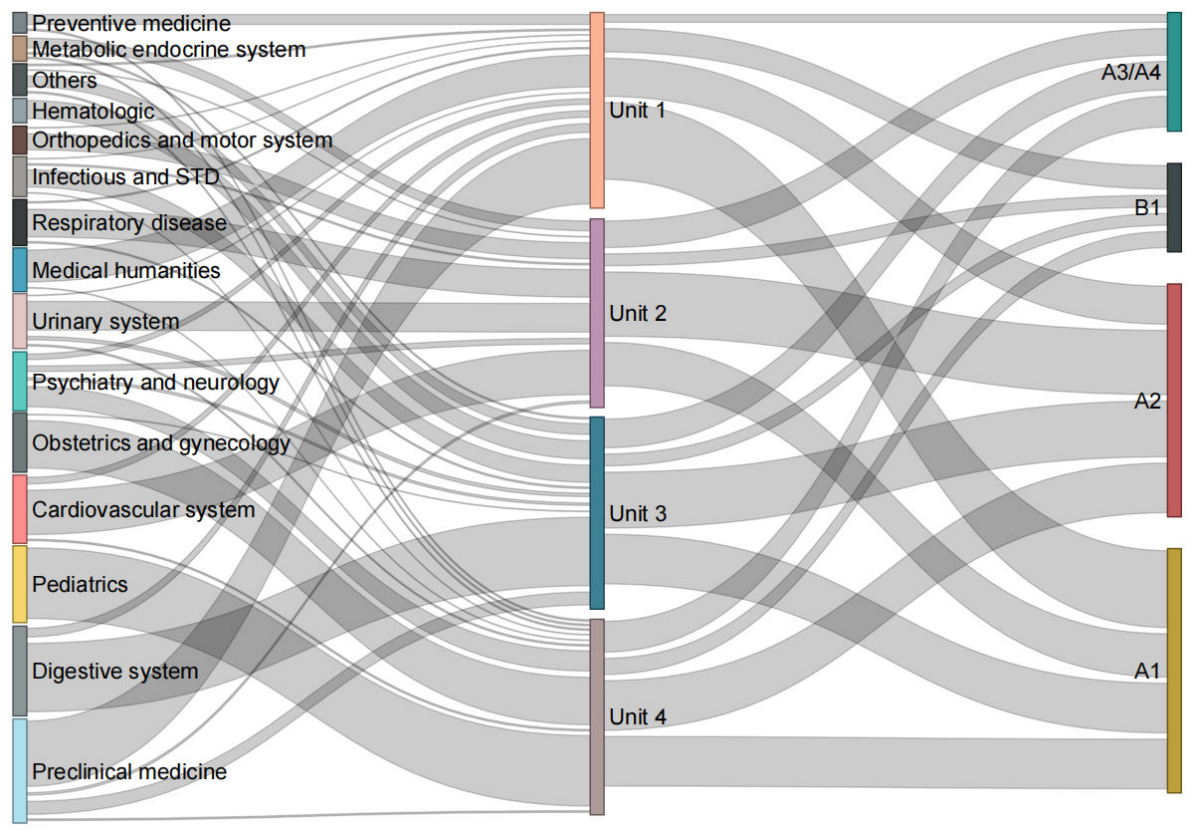
The Sankey diagram of the 3 question classifications: the medical subspecialties, units, and types. STD: sexually transmitted disease.

According to the introduction of the Chinese NMEC [22], each examination unit always addresses specific medical subspecialties. Unit 1 covers medical knowledge, policies, regulations, and preventive medicine; unit 2 mainly pertains to the cardiovascular, urinary, musculoskeletal, and endocrine systems; unit 3 involves the digestive, respiratory, and associated systems; unit 4 focuses on obstetrics and gynecology, pediatrics, and neurological or psychiatric domains. However, such distribution is not absolute. Therefore, 2 clinicians independently reclassified the 600 questions into 15 medical subspecialties, resolving discrepancies through discussion. The κ value for the result of their classifications was 0.935. The Sankey diagram of the 3 question classifications, medical subspecialties, units, and types is shown in Figure 1.

### Instructions Before Testing Part

Before manually inputting questions, ChatGPT was informed about an upcoming series of queries. ChatGPT needed to identify the most plausible response from the available options and explain the reasoning behind its selection. The question types determined the relevant lead-in prompts provided. For the A1 and A2 question types, each input question was deemed independent, rendering any interquestion relationships irrelevant. In contrast, A3/A4 question types implied that multiple questions within a single clinical case shared a connection. However, individual clinical cases were treated as discrete entities, eliminating the need to consider relationships between them. For the B1 question type, 5 shared options were given. ChatGPT needed to identify the correct answers for subsequent questions. Chaining was used in A3/A4 and B1 question types to ensure that multiple questions within a single clinical case in A3/A4 shared the same context, and multiple questions in B1 shared the same options. The number of questions inputted at one time depended on the text’s length, such as 5‐8 questions for A1/A2 types. If necessary, ChatGPT was forced to disregard prior conversational content and commence a fresh chat.

### Temperature

The temperature parameter in ChatGPT influences the randomness of the model’s responses. A higher temperature yields more varied and creative answers. In our study, we did not manually adjust the temperature; instead, we used the default setting on the OpenAI platform, commonly at 0.7, to simulate real-world user interactions on the front end. This balance between typical user habits and diverse thought processes was intentional. The default relatively high temperature was expected to enable ChatGPT to generate more creative reasoning processes while still arriving at accurate answers.

### Testing Strategy

All the CNMLE 2022 questions were tested in Chinese according to the following 2 factors:

1. ChatGPT model selection. Both GPT-3.5 (version from March 23) and GPT-4.0 (version from May 3) were rigorously evaluated on the OpenAI platform from April 24 to May 15, 2023, to ascertain any evolution in the model’s capability in the medical domain.
2. System role. This refers to the specific identity or role, such as “gastroenterology specialist,” assigned to ChatGPT to determine if relevant knowledge is applied more accurately. Questions were evaluated both with and without assigning a system role related to the 15 specific clinical subspecialties. This system role was designated by providing a tailored system prompt before the testing instructions, aiming to guide ChatGPT’s approach and align it with specialist viewpoints in the relevant medical field.

### Testing Process

Considering the evaluation of the ChatGPT model, system role, and response coherence, each question was tested 8‐12 times. The prompts included those specific to question types, the assignment of system role, and the use of chaining. Slight modifications in these prompts were adopted to avoid potential systematic errors introduced by rigid wording. For example, the prompt “Assume you are a gastroenterology specialist” might vary as “Assume you are highly proficient in gastroenterology.” For coherence evaluation, each question was presented again to ChatGPT. If the regenerated response matched the initial answer, the process was halted. However, if the 2 responses differed, the question was posed once more to ChatGPT.

### Response Determination

The first and second responses from ChatGPT were directly assessed against the given standard answers for accuracy. For the final response (referred to as joint response), if 2 of the 3 answers were consistent, this was taken as the conclusive answer and evaluated against the standard. However, if the 3 responses were all distinct, it was automatically marked as incorrect without any further comparison to the standard answer.

The first response was more applicable to assessing whether ChatGPT could pass the CNMLE in the same situation as a student examinee. In contrast, the joint response represented an overall accuracy (the proportion of questions answered correctly at least twice) [17], which was more suitable for demonstrating the potential of ChatGPT in medical education.

According to the announcement from the CNMLE Committee of the National Health Commission of China, the passing score for licensed physicians is 360 points, which means an accuracy rate of 60% or above is considered a pass.

### Statistical Analysis

Data were collected and managed using Excel software. The statistical analyses were conducted with SPSS (version 26.0.0; IBM Corp). A *χ*^2^ test was used to compare the accuracy of CNMLE question responses between different testing strategies and subgroups of question types. Variability was calculated by the number of consistently correct or wrong answers in 2 repeated responses divided by the total number of questions (600). Additionally, the κ statistic was used to evaluate answer consistency. A difference was considered statistically significant when *P*<.05.

### Ethical Considerations

This study collected information that was already published in the bookstore and did not involve human subjects; therefore, approval by the Institutional Review Board of Henan Provincial People’s Hospital was not required.

## Results

### Accuracy and System Role Assignment

In model comparison, GPT-3.5 achieved an initial accuracy of 54% (324/600) and did not meet the exam criteria. Conversely, GPT-4.0 achieved a passing accuracy of 72.7% (436/600), which was significantly higher than GPT-3.5 (*P*<.001). Similarly, with a designated system role, GPT-4.0 still exhibited higher accuracy than GPT-3.5 (73% vs 55.3%; *P*<.001).

Upon system role assignment, both GPT-3.5 and GPT-4.0 showed a slight increase in accuracy compared to when no role was assigned; specifically, 55.3% (332/500) from 54% (324/600) for GPT-3.5 (*P*>.05) and 73% (438/600) from 72.7% (436/600) for GPT-4.0 (*P*>.05).

The upper comparisons for the second and joint responses paralleled the initial results, as shown in Table 1.

**Table 1.** Accuracy of GPT-4.0 and 3.5 with or without SR designation under repeat tests. n represents the number of correct answers.

Accuracy	GPT-3.5, n (%)	GPT-4.0, n (%)	*P* value	GPT-3.5 + SR^a^, n (%)	GPT-4.0 + SR, n (%)	*P* value
IR^b^	324 (54.0)	436 (72.7)	<.001	332 (55.3)	438 (73.0)	<.001
2R^c^	303 (50.5)	426 (71.0)	<.001	310 (51.7)	448 (74.7)	<.001
JR^d^	302 (50.3)	435 (72.5)	<.001	329 (54.8)	437 (72.8)	<.001

a SR: system role.

b IR: initial response.

c 2R: second response.

d JR: joint response.

### Variability of Responses

The GPT-3.5 model exhibited a variability rate of 19.5% (117/600), which decreased to 17.7% (106/600) upon the designation of a system role. The variability rate for GPT-4.0 was observed at 9% (54/600), and further reduced to 7.3% after a system role was assigned. These results indicated a smaller response variability for GPT-4.0 compared to GPT-3.5, and specifying system roles also decreased the variability rates. Both models showed relatively high coherence between the initial and second response, with κ values of 0.778 and 0.610. Detailed information for repeated response can be seen in Multimedia Appendix 2.

### Accuracy for Subgroups

For GPT-4.0, when accounting for system role and repeated responses, there was a statistically significant difference in accuracy across the different units for the CNMLE test, with accuracy ranging from 62% (93/150) to 84% (126/150; *P* range from<.001 to .01). However, when grouped by question type, the accuracy ranged from 69.4% (145/209) to 83.1% (59/71) without statistical difference (*P*>.28).

In contrast, for GPT-3.5, only the initial response with system role designation showed a statistical difference in accuracy (*P*=.04) for question type subgroups. In other groupings by unit or question type, as well as in subsequent responses, the accuracy remained without significant variations (*P*>.14; see Table 2).

**Figure 2.**
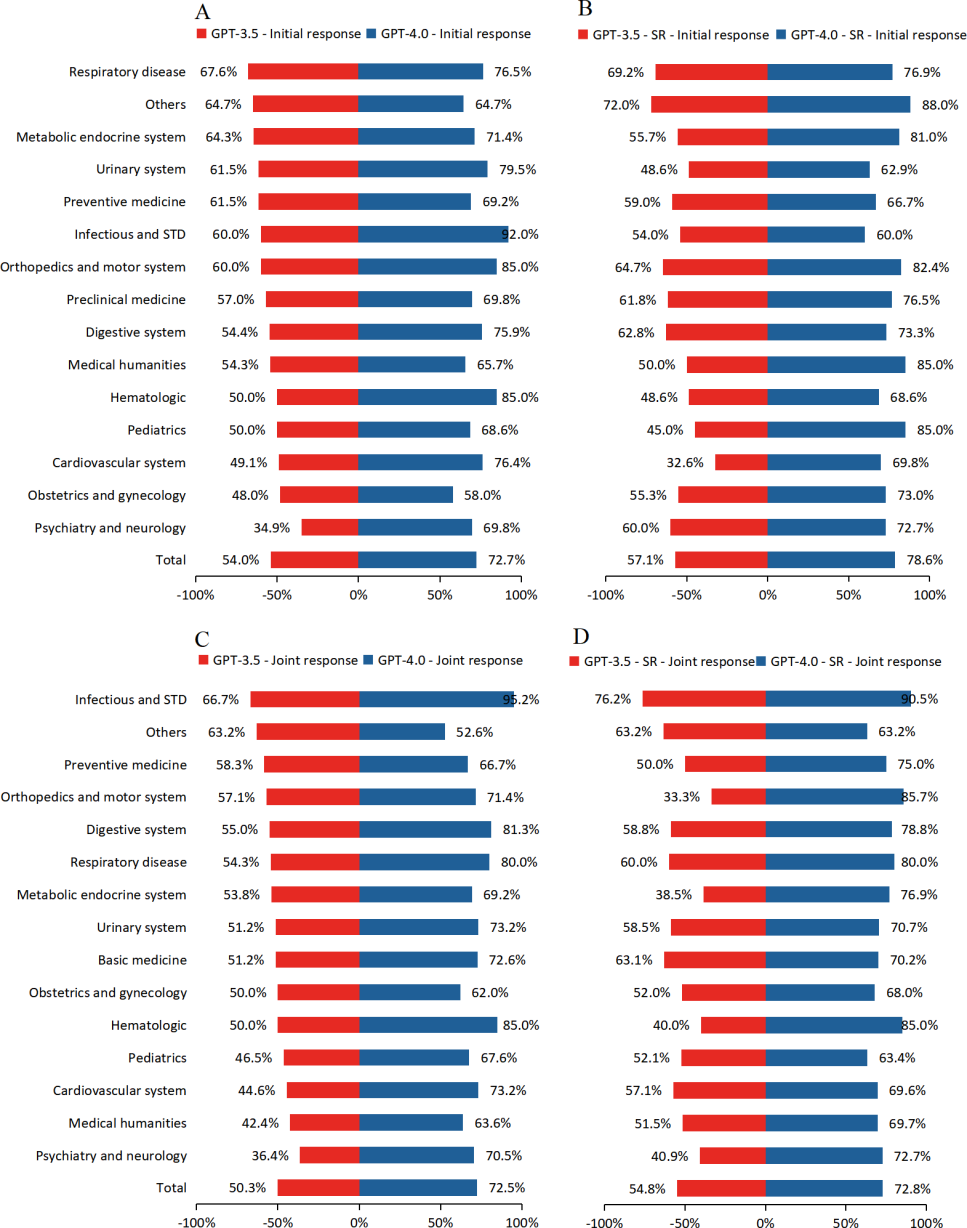
Accuracy for GPT-3.5/4.0 classified by 15 medical subspecialties. (A) the initial response, (B) the initial response with SR assignation, (C) the joint response, (D) the joint response with SR assignation. SR: system role; STD: sexually transmitted disease.

Accuracy for initial and joint responses of GPT-3.5/4.0 classified by 15 medical subspecialties is shown in Figure 2. In multiple testing strategies, GPT-4.0 outperformed GPT-3.5 in accuracy for 14 distinct clinical subspecialty questions, consistently surpassing the 60% passing threshold.

**Table 2.** Subgroup analysis of accuracy for the 4 sections and 4 question types under different strategies. Data were showed as n (%). Units 1‐4 were the 4 parts to which the questions belonged, and A1-A2, B1 represented the types of questions. Units 1‐4 corresponded to distinct clinical subspecialties, with specific details provided in the Methods section.

Model strategy	Unit 1(n=150), n (%)	Unit 2(n=150), n (%)	Unit 3(n=150), n (%)	Unit 4(n=150), n (%)	*P* value	A1(n=220), n (%)	A2(n=209), n (%)	A3/A4(n=100), n (%)	B1(n=71), n (%)	*P* value
GPT3.5: IR^a^	82 (54.7)	83 (55.3)	88 (58.7)	71 (47.3)	.25	122 (55.5)	109 (52.2)	59 (59.0)	34 (47.9)	.47
GPT3.5: 2R^b^	71 (47.3)	77 (51.3)	85 (56.7)	70 (46.7)	.28	115 (52.3)	103 (49.3)	57 (57.0)	28 (39.4)	.14
GPT3.5: JR^c^	72 (48.0)	75 (50.0)	86 (57.3)	69 (46.0)	.22	114 (51.8)	101 (48.3)	57 (57.0)	30 (42.3)	.24
GPT3.5: IR+ SR^d^	85 (56.7)	84 (56.0)	91 (60.7)	72 (48.0)	.16	129 (58.6)	113 (54.1)	61 (61.0)	29 (40.8)	.04
GPT3.5: 2R + SR	83 (55.3)	74 (49.3)	82 (54.7)	71 (47.3)	.42	121 (55.0)	102 (48.8)	57 (57.0)	30 (42.3)	.15
GPT3.5: JR+ SR	84 (56.0)	80 (53.3)	91 (60.7)	74 (49.3)	.25	126 (57.3)	110 (52.6)	61 (61.0)	32 (45.1)	.16
GPT4.0: IR	102 (68.0)	118 (78.7)	119 (79.3)	97 (64.7)	.006	154 (70.0)	152 (72.7)	79 (79.0)	51 (71.8)	.42
GPT4.0: 2R	100 (66.7)	112 (74.7)	119 (79.3)	95 (63.3)	.009	155 (70.5)	145 (69.4)	76 (76.0)	50 (70.4)	.68
GPT4.0: JR	104 (69.3)	114 (76.0)	121 (80.7)	96 (64.0)	.007	157 (71.4)	146 (69.9)	79 (79.0)	53 (74.6)	.37
GPT4.0: IR+ SR	103 (68.7)	116 (77.3)	126 (84.0)	93 (62.0)	<.001	157 (71.4)	151 (72.2)	72 (72.0)	58 (81.7)	.37
GPT4.0: 2R + SR	104 (69.3)	117 (78.0)	124 (82.7)	103 (68.7)	.01	159 (72.3)	153 (73.2)	77 (77.0)	59 (83.1)	.28
GPT4.0: JR+ SR	101 (67.3)	115 (76.7)	124 (82.7)	97 (64.7)	.001	156 (70.9)	151 (72.2)	73 (73.0)	57 (80.3)	.47

a IR: initial response.

b 2R: second response.

c JR: joint response.

d SR: system role.

## Discussion

### Overview

The CNMLE syllabus outlines the essential knowledge and competencies that physicians need for diagnostic and therapeutic procedures. Acquiring these competencies typically demands that a medical student invest several years in both theoretical education and practical skill development. The application of ChatGPT in medical examinations, particularly within the CNMLE framework, offers a pioneering approach to gauge the potential of LLMs in clinical diagnosis and treatment planning. This study comprehensively assessed ChatGPT’s performance in addressing CNMLE questions, focusing on model evolution and system role designation, which has not yet been fully investigated.

### Model Evolution and Performance

In our study, GPT-4.0 consistently outperformed GPT-3.5 in accuracy and reliably met the passing criteria set by the CNMLE Committee. Despite GPT-3.5 achieving an accuracy rate of over 50%, it failed to pass the examination. A noncomparison study using GPT-3.5 to test CNMLE 2020‐2022 achieved an accuracy of (36.5%‐47%) [23]. The lower accuracy might be attributed to the fact that the testing was conducted before February, shortly after the release of GPT-3.5. The better performance of GPT-4.0 compared with GPT-3.5 was also reported by Wang et al [24]. However, it is noteworthy that their assessment was based on a limited sample of 100 questions, rather than a full set of 600 questions. The small sample might have contributed to the overall favorable results (GPT-4.0: 84%; GPT-3.5: 56%). Therefore, our findings might provide a more representative comparison of the real-world performance of GPT-4.0 and 3.5 on the CNMLE.

Other research on evaluating ChatGPT’s accuracy on national medical licensing examinations included assessments of the USMLE [1,2] and the Japanese National Medical Licensing Examination [7]. The conclusions were similar to ours: while GPT-3.5 was often at or near the passing threshold, GPT-4.0 passed relevant exams and had higher testing accuracy compared to GPT-3.5. This trend was not only limited to national medical licensing examinations but also applied to other medical-related examinations. However, the specific accuracy varied across models, possibly due to differences in study countries, testing time, exam content, and other variables. A comprehensive review of existing published and non–peer-reviewed research findings is available in Table 3.

**Table 3.** A review of the existing published and non–peer-reviewed research related to ChatGPT performance on medical examinations.

Study	Country	Test model	Examination	Data sample, n	Passing threshold	Accuracy (%)
Gilson et al [1]	United States	GPT-3.5	The United States Medical Licensing Examination Step 1 and Step 2 exams	87‐102	60%	GPT-3.5: 44.0‐64.4
Kung et al [2]	United States	GPT-3.5	The United States Medical Licensing Exam	376	60%	At or near 60%
Guerra et al [25]	United States	GPT-4.0 and 3.5	Congress of Neurological Surgeons Self-Assessment Neurosurgery Exam	591	—^a^	GPT-4.0: 76.6; GPT-3.5: 60.2
Takagi et al [7]	Japan	GPT-4.0 and 3.5	Japanese National Medical Licensing Examination (2023)	254	GPT-4.0: Pass; GPT-3.5: Failed	GPT-4.0: 79.9; GPT-3.5: 50.8
Wang et al [24]	China	GPT-4.0 and 3.5	The Chinese National Medical LicensingExamination	100	—	GPT-4.0: 84; GPT-3.5: 56
Cai et al [26]	United States	GPT-4.0 and 3.5	Ophthalmology Board-Style Questions	250	—	GPT-4.0: 71.6; GPT-3.5: 58.8
Oh et al [8]	Korea	GPT-4.0 and 3.5	Korean General Surgery Board Exams	280	—	GPT-4.0: 76.4; GPT-3.5: 46.8
Skalidis et al [27]	Switzerland	GPT-3.5	The European Exam in Core Cardiology	488	Pass	GPT-3.5: 58.8
Saad et al [28]	United Kingdom	GPT-4.0	The Orthopaedic FRCS Orth Part A exam	240	Failed	GPT-4.0: 67.5
Weng et al [5]	China	GPT-3.5	Taiwan’s 2022 Family Medicine Board Exam	125	Failed	GPT-3.5: 41.6
Kumah-Crystal et al [29]	United States	GPT-3.5	The Clinical Informatics Board Examination	254	60%, Pass	GPT-3.5: 74
Mihalache et al [30]	Canada	GPT-4.0	OphthoQuestions practice question bank for board certification examination	125	—	GPT-4.0: 84
Ali et al [31]	United States	GPT-4.0 and 3.5	Self-Assessment Neurosurgery Examination Indications Examination	149	—	GPT-4.0: 82.6; GPT-3.5: 62.4
Oztermeliet al [32]	Turkey	GPT-3.5	Turkey Medical Specialty Exams	1177	—	GPT-3.5: 54.3‐70.9
Fijaoko et al [4]	United States	GPT-3.5	American Heart Association Basic Life Support and Advanced Cardiovascular Life Support exams	126	84%, Failed	GPT-3.5: 64‐68.4
Su et al [12]	China (Taiwan)	GPT-3.5	Taiwan's 2022 Nursing Licensing Exam	400	Pass	GPT-3.5: 80.75%
Lewandowski et al [33]	Poland	GPT-4.0 and 3.5	The Dermatology Specialty Certificate Examinations	120 × 3	GPT-4 Pass	GPT-4.0: >70% better than GPT-3.5
Kung et al [34]	United States	GPT-4.0 and 3.5	Orthopaedic In-Training Examination (2020‐2022)	360	GPT-4.0: >PGY^b^-5 level; GPT-3.5: PGY-1 level	GPT-4.0: 73.6; GPT-3.5: 54.3
Gencer and Aydin [35]	Turkey	GPT-4.0 and 3.5	Turkish-language thoracic surgery exam	105	Surpass students’ scores	GPT-4.0: 93.3; GPT-3.5: 90.5

a Not available.

b PGY: postgraduate year.

### System Role for Accuracy

While it was expected that introducing system role tailored for clinical subspecialties would enhance the reliability of ChatGPT’s medical responses, this effect had not been systematically studied. Our research addressed this gap. Our findings revealed slight but noteworthy improvements in accuracy for both GPT-3.5 (1.3%‐4.5%) and GPT-4.0 (0.3%‐3.7%), although these gains were not statistically significant. This might imply that ChatGPT’s inherent abilities are already robust enough to discern and address the medical inquiries without narrowing down its response scope.

### Response Variability

As an LLM, ChatGPT naturally exhibits variability in responses when the temperature hyperparameter is not zero. In this study, we adopted the default temperature of 0.7 to simulate real-world use conditions on the front end. Our results showed relatively high coherence between the initial and second responses for both GPT-4.0 and GPT-3.5. Therefore, the relatively high temperature of 0.7 is feasible and recommended when testing ChatGPT’s performance on the CNMLE. Furthermore, our results highlighted that both model evolution and system roles contribute to ChatGPT’s variability in scenarios such as the Chinese Medical Licensing Exams. This variability can be valuable for medical education, as ChatGPT not only provides answers to questions but also includes the rationale and references for its choices, which allows students to easily follow and comprehend [16]. Repeatedly submitting questions allows groups or individuals to engage with the explanatory content generated by ChatGPT, which is particularly beneficial for open-ended case scenario discussions [17].

### Subgroup and Multispecialty Analysis

Our subgroup analysis revealed that ChatGPT demonstrated consistent accuracy across different types of questions. This indicated that ChatGPT was capable of understanding and analyzing complex medical cases and scenarios (A2, A3/A4 questions), which can be challenging even for humans, and making correct decisions. This decision-making ability was equally proficient when addressing more straightforward, common-sense questions that did not require reasoning (A1, B1 questions).

In comparisons among unit subgroups representing different subspecialties, significant performance variations were observed in GPT-4.0 across CNMLE units. GPT-4.0 exhibited higher accuracy in units 2‐3, which predominantly featured questions from subspecialties such as cardiovascular, urinary, digestive, and respiratory systems. This was further corroborated by our multispecialty analysis results. GPT-4.0 achieved an accuracy rate of over 75% for these 4 subspecialties, surpassing its overall accuracy rate of 72.7%. Given that these 4 subspecialties accounted for a substantial proportion (34.5%) of all 15 subspecialties, such a disparity might have been advantageous. However, this disparity disappeared upon the introduction of system roles as prompts, with the overall accuracy of GPT-4.0 increasing to 78.6%. This might suggest that the appropriate use of system roles could compensate for individual subspecialty question accuracy, thereby enhancing the overall accuracy of ChatGPT.

Furthermore, we used CNMLE questions, divided into 15 medical subspecialties, to comprehensively assess the medical expertise of ChatGPT models. This approach provided a robust framework for evaluating model proficiency across a variety of medical fields. Notably, GPT-4.0 surpassed the 60% passing threshold in 14 of the 15 distinct clinical subspecialties, in contrast to GPT-3.5, which only passed in 7 out of 15 subspecialties. This highlighted the superiority of GPT-4.0 and its potential in medical applications.

### Generalizability of Findings

Previous studies [7] often excluded table and image-based questions when evaluating ChatGPT’s performance in medical exams. This approach limited the generalizability of these findings due to ChatGPT’s lack of multimodal data processing. In contrast, our study, focusing on the CNMLE’s multiple-choice format, which almost exclusively consists of nongraphical and nontabular questions, offers greater generalizability in real exam settings. Zhu et al [17] suggested that ChatGPT, as a chatbot, had advantages in responding to open-ended questions, corresponding more closely with real-world scenarios where users sought medical support knowledge from ChatGPT. The potential of ChatGPT in exams with open-ended questions merits further exploration.

### Limitations

First, this study assessed ChatGPT’s ability to answer questions from the Chinese version of the CNMLE. As ChatGPT is mainly trained on English data, Chinese questions could have underestimated its capabilities. Second, the CNMLE questions were multiple-choice, introducing the chance factor in selecting correct answers. Limited by paper length, we did not evaluate the logic behind ChatGPT’s choices, although this aspect is critical and merits deeper investigation. Third, real-world medical questions often have open-ended, multiple, or uncertain answers. Therefore, the CNMLE may not represent the full scope of challenges ChatGPT might face in clinical settings. Consequently, GPT-4.0’s success on the CNMLE may only indicate its partial competence in clinical decision-making. Future studies should broaden the range of question types to better assess ChatGPT’s medical performance. Despite these limitations, we believe this study provided valuable insights into ChatGPT’s capabilities in medicine.

### Conclusions

This study comprehensively evaluated the performance of GPT-4.0 and GPT-3.5 in the context of the CNMLE. Our findings indicated that GPT-4.0 not only met the CNMLE passing criteria but also significantly outperformed GPT-3.5 in key areas such as accuracy, consistency, and medical subspecialty expertise. Furthermore, the implementation of system roles served as a pivotal factor in enhancing the model’s reliability and answer coherence. These results collectively underscored GPT-4.0’s promising potential as a valuable tool for medical professionals, educators, and students, warranting further research and application in the medical field.

## Supplementary material

10.2196/52784 Multimedia Appendix 1 Question type explanations conveyed to ChatGPT via structured prompts.

10.2196/52784 Multimedia Appendix 2 Detail information for repeated responses and their κ value under the ChatGPT default temperature of 0.7.

## Abbreviations

CNMLE: Chinese National Medical Licensing Examination
LLM: large language model
NMEC: National Medical Examination Center
USMLE: United States Medical Licensing Examination
