# Supplementary material for: Influence of Model Evolution and System Roles on ChatGPT’s Performance in Chinese Medical Licensing Exams: Comparative Study
Source: JMIR Med Educ. 2024 Aug 13;10:e52784. doi: 10.2196/52784 (PMC11336778; doi:10.2196/52784)
Supplement: Multimedia Appendix 1 [file mededu-v10-e52784-s001.docx]

**Supplemental S1.**

| **Prompts for Question Types** | **Official Introductions of Chinese version** | **Translation for the Introductions** |
| --- | --- | --- |
| A1 | 每一道考题下面有A、B、C、D、E五个备选答案，请从中选择一个最佳答案 | Each question is followed by five optional answers: A, B, C, D, and E. Please choose the best answer from them. |
| A2 | 每一道考题是以一个小案例出现的，其下面都有A、B、C、D、E五个备选答案，请从中选择一个最佳答案 | Each question is presented as a mini-case, followed by five optional answers: A, B, C, D, and E. Please choose the best answer from them. |
| A3/A4 | 以下提供若干个案例，每个案例下设若干道试题。请根据案例提供的信息，在每一道题下面的A、B、C、D、E五个备选答案中选择一个最佳答案 | Below are several case studies, with multiple questions set under each case. Based on the information provided in the case, please choose the best answer from the five options A, B, C, D, and E provided under each question |
| B1 | 以下提供若干组考题，每组考超共用在考题前列止的A、B、C、D、E五个备选答案，请从中选择一个与同题关系最密切的答案，每个备选答案可能被选一次，多次或者不被选择。 | Below are several groups of questions. For each group, there are five options A, B, C, D, and E listed before the questions. Please choose the answer most closely related to the question from these options. Each option may be chosen once, multiple times, or not at all |
