# Supplementary material for: Influence of Model Evolution and System Roles on ChatGPT’s Performance in Chinese Medical Licensing Exams: Comparative Study
Source: JMIR Med Educ. 2024 Aug 13;10:e52784. doi: 10.2196/52784 (PMC11336778; doi:10.2196/52784)
Supplement: Multimedia Appendix 2 [file mededu-v10-e52784-s002.docx]

Supplemental S2: Detail information for repeated responses and their Kappa under the ChatGPT default temperature of 0.7

Table 1. The Kappa values and paired χ2 test of the initial and 2nd responses for GPT-4.0 and 3.5.

| Test w/o SR | | GPT-3.5 - 2^nd^R | | Kappa | *P* | GPT-4.0 - IR | | Kappa | P |
| --- | --- | --- | --- | --- | --- | --- | --- | --- | --- |
|  |  | W | R |  |  | W | R |  |  |
| GPT-3.5 - IR | W | 228 | 48 | 0.610 | 0.064 | 121 | 155 | 0.315 | < 0.001 |
|  | R | 69 | 255 |  |  | 43 | 281 |  |  |
| GPT-4.0 - 2^nd^R | W | 131 | 166 | 0.300 | < 0.001 | 142 | 22 | 0.778 | 0.220 |
|  | R | 43 | 260 |  |  | 32 | 404 |  |  |

Note: Data was showed as 2*2 cross table. SR: system role. W/o: without. IR: initial response. 2ndR: The 2nd response. JR: joint response.

Table 2. The Kappa values and paired χ^2^ test of the initial and 2nd responses for GPT-4.0 and 3.5 with SRs designation.

| Test with SR | | GPT-3.5 - 2^nd^R | | Kappa | *P* | GPT-4.0 - IR | | Kappa | P |
| --- | --- | --- | --- | --- | --- | --- | --- | --- | --- |
|  |  | W | R |  |  | W | R |  |  |
| GPT-3.5 - IR | W | 226 | 42 | 0.645 | 0.041 | 117 | 151 | 0.313 | < 0.001 |
|  | R | 64 | 268 |  |  | 45 | 287 |  |  |
| GPT-4.0 - 2^nd^R | W | 115 | 175 | 0.282 | < 0.001 | 135 | 27 | 0.810 | 0.174 |
|  | R | 37 | 273 |  |  | 17 | 421 |  |  |

Note: Data was showed as 2*2 cross table. SR: system role. W/o: without. IR: initial response. 2ndR: The 2nd response. JR: joint response.
